# The Mitochondrial Translocator Protein and the Emerging Link Between Oxidative Stress and Arrhythmias in the Diabetic Heart

**DOI:** 10.3389/fphys.2018.01518

**Published:** 2018-10-26

**Authors:** Zeki Ilkan, Fadi G. Akar

**Affiliations:** Cardiovascular Research Center, Icahn School of Medicine at Mount Sinai, New York, NY, United States

**Keywords:** arrhythmias, reactive oxygen species, oxidative stress, mitochondria, diabetes

## Abstract

The mitochondrial translocator protein (TSPO) is a key outer mitochondrial membrane protein that regulates the activity of energy-dissipating mitochondrial channels in response to oxidative stress. In this article, we provide an overview of the role of TSPO in the systematic amplification of reactive oxygen species (ROS) through an autocatalytic process known as ROS-induced ROS-release (RIRR). We describe how this TSPO-driven process destabilizes the mitochondrial membrane potential leading to electrical instability at the cellular and whole heart levels. Finally, we provide our perspective on the role of TSPO in the pathophysiology of diabetes, in general and diabetes-related arrhythmias, in particular.

## Introduction

**FIGURE 1:**
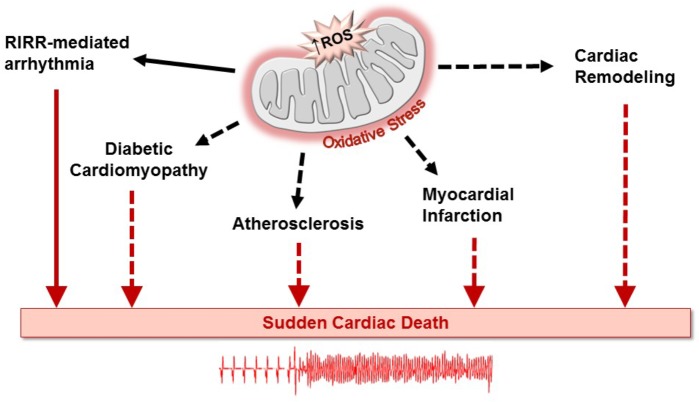
Oxidative stress created by excessive generation of mitochondrial reactive oxygen species (ROS) plays well-recognized roles in the development of an array of cardiovascular disorders leading to sudden cardiac death. ROS production can be amplified through the process of ROS-induced ROS release (RIRR), causing cellular dysfunction and death, directly creating a pro-arrhythmic substrate (solid arrows). In a relatively more indirect manner, ROS production can increase the risk of lethal arrhythmias by promoting adverse remodeling of various pathways in the diabetic heart (dashed arrows) (Wilson et al., 2018). Similarly, ROS production can contribute to the progression of atherosclerosis and myocardial infarction as well as to heart failure-associated cardiac remodeling, increasing the susceptibility of the heart to arrhythmias that can result in sudden cardiac death.

Diabetes mellitus is a global public health epidemic that continues to expand, in both its incidence and prevalence. Diabetic patients are predisposed to an increasing number of debilitating cardiovascular disorders such as stroke and myocardial infarction (Aune et al., 2018; Yang et al., 2018). This metabolic disease is also an important risk factor in the development of cardiac rhythm disorders (Movahed et al., 2005; Huxley et al., 2012; Lau et al., 2017). In addition to predisposing to atrial fibrillation (Huxley et al., 2011), diabetes along with its numerous complications, has been linked to increased prevalence of ventricular arrhythmias leading to sudden cardiac death (Stahn et al., 2014; Pistrosch et al., 2015; Xie et al., 2015; Agarwal and Singh, 2017). Importantly, oxidative stress, a major factor in the pathophysiology of diabetes, has been linked to arrhythmias either directly or through exacerbation of atherogenic risk factors (Van Wagoner, 2008; Gutierrez and Van Wagoner, 2015). Oxidative stress arises from enhanced production of free radicals and defective antioxidant defense mechanisms in the diabetic heart (Bashan et al., 2009; Bajaj and Khan, 2012). This, in turn, contributes to the pathogenesis of numerous diabetes-related cardiovascular complications, including endothelial dysfunction (Higashi et al., 2009), atherosclerosis (Harrison et al., 2003), myocardial infarction (Misra et al., 2009), and diabetic cardiomyopathy (Jia et al., 2018), all of which as illustrated in Figure 1 can lead to sudden cardiac death (Anderson et al., 2009; Duicu et al., 2016; Peiro et al., 2016; Zhang et al., 2017). In this article, we focus on a key outer mitochondrial membrane protein known as the mitochondrial translocator protein (TSPO) as a source of oxidative stress-related cardiac dysfunction. We begin by highlighting its role in linking mitochondrial instability to arrhythmias in the heart through a regenerative process known as reactive oxygen species (ROS) -induced ROS-release (RIRR). We then provide a new perspective on its potential importance to the pathophysiology of diabetes, in general and diabetes-related arrhythmias, in particular.

## The Mitochondrial Translocator Protein

**FIGURE 2:**
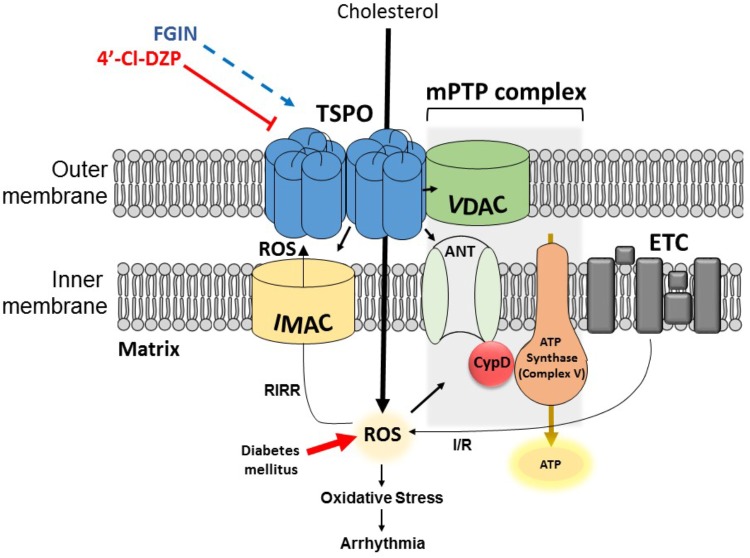
Simplified illustration of a TSPO dimer, IMAC, and the mPTP complex on the mitochondrial membrane. TSPO is located on the outer mitochondrial membrane and facilitates cholesterol transport into the matrix. The close apposition of TSPO with IMAC and mPTP allows it to carry out its modulatory role in processes such as RIRR. During normal mitochondrial respiration, electrons which escape the ETC can combine with oxygen forming O^-^_2_ anions. ROS-scavenging enzymes work toward removing the ROS and hence keeping the cells healthy. Excessive production and/or defective scavenging of ROS under pathological conditions such as I/R and/or diabetes mellitus can activate ROS-sensitive IMAC, which amplifies ROS levels via RIRR. Escalating oxidative stress then activates the mPTP complex which can result in ΔΨ_m_ depolarization, leading to mitochondrial dysfunction, ischemic injury, and electrical remodeling paving way to arrhythmia. Additionally, internalized cholesterol can become oxidized by accumulating ROS, generating oxysterols which further enhance oxidative stress. The most common TSPO ligands 4′-chlorodiazepam (4′-Cl-DZP) and FGIN-1-27 have been used by many studies to study the involvement of TSPO in these processes.

TSPO, formerly known as the peripheral benzodiazepine receptor (PBR) (Papadopoulos et al., 2006), is a structurally conserved molecule which is ubiquitously expressed in steroidogenic tissues, as well as brain, kidney, and heart cells (Rupprecht et al., 2010; Morin et al., 2016). It was discovered in 1977, and initially called PBR because of its ability to bind benzodiazepine drugs outside of the central nervous system (Braestrup and Squires, 1977). The 18-kDa molecule carries out a variety of essential roles such as cholesterol transport (Li and Papadopoulos, 1998), steroidogenesis (Besman et al., 1989; Papadopoulos et al., 1997), and programmed cell death (Caballero et al., 2013). In eukaryotes TSPO is mainly expressed on the outer mitochondrial membrane, in close physical association with other mitochondrial channels such as the voltage-dependent anion channel (VDAC) within the mitochondrial membrane transition pore (mPTP) complex, and the inner membrane anion channel (IMAC) (Veenman et al., 2007; Motloch et al., 2015) (Figure 2). Cryo-electron microscopy and image analyses of the TSPO molecule from *Rhodobacter sphaeroides* revealed a dimeric quaternary structure, whereby each TSPO monomer consists of five transmembrane domains (Korkhov et al., 2010; Li et al., 2015). Although monomeric and oligomeric forms have been reported, the functional implications of TSPO polymerization have not been fully elucidated (Lacapere et al., 2001; Delavoie et al., 2003; Jaremko et al., 2014). The revelation of the 3-dimensional high-resolution image of the mouse TSPO has provided the opportunity to study the molecular interactions between this mitochondrial protein and its ligands, such as the antagonist PK11195 (Jaremko et al., 2014). The functional role of TSPO in various organs and cell types has been investigated primarily using TSPO agonists and antagonists (Fulda et al., 2010; Rupprecht et al., 2010). In order to fully appreciate the role of TSPO as a mediator of cardiac pro-arrhythmic risk, we begin by reviewing the concept of RIRR which directly links mitochondrial instability to myocyte excitability.

## Ros-Induced Ros-Release and Mitochondrial Instability as a Mediator of Cardiac Arrhythmias

Mitochondria have long been recognized as indispensable sources of adenosine triphosphate (ATP) in energy-reliant organs such as the heart. Almost counterintuitively, it later became apparent that these specialized organelles can also control cell death in response to injury. In healthy mammalian cells, the preservation of ATP synthesis by complex V is achieved by maintaining a proton gradient across the inner mitochondrial membrane (Mitchell and Moyle, 1965a,b), which in turn, generates an electrochemical gradient that is responsible for maintaining a polarized mitochondrial membrane potential (Kroemer et al., 2007). Mitochondrial respiration is always accompanied with ROS production through leakage of electrons that subsequently react with oxygen to form superoxide anions (O^-^_2_) (Turrens, 2003). Under certain pathological conditions such as diabetes, the production of ROS can exceed the capacity with which protective antioxidant defense systems eliminate these toxic agents. Oxidative stress, as well as secondary factors such as mitochondrial Ca^2+^ overload can prime the formation of mPTP on the inner mitochondrial membrane (Zorov et al., 2000; Aon et al., 2003, 2006, 2007; Halestrap and Pasdois, 2009). This is responsible, at least in part, for mitochondrial membrane permeabilization (Green, 2005), which can be underpinned by the process of RIRR (Zorov et al., 2000; Aon et al., 2003, 2006; Brady et al., 2006; Yang et al., 2010). Traditionally, mPTP has been thought to exist as a complex of proteins comprising of VDAC, adenine nucleotide translocator (ANT), and cyclophilin D (CypD) (Figure 2). Nevertheless, genetic studies in recent years have challenged this traditionally accepted model of mPTP structure. For more details on this subject matter, we refer the reader to another review (Kwong and Molkentin, 2015).

Sollot and colleagues (Zorov et al., 2000) pioneered the concept of RIRR to describe how ROS injuries confined to distinct areas of a cardiomyocyte are able to quickly spread through a wider network of mitochondria, culminating in oxidative stress at a cellular level (Zorov et al., 2006). RIRR is responsible for the autocatalytic amplification of ROS levels, eventually leading to cell death. Two modes of RIRR have been proposed based on the identity of the mitochondrial pathway that mediates the process, namely the mPTP or the IMAC (Brady et al., 2006; Yang et al., 2010). Initially, the connection between mPTP opening and oxidative stress-dependent destabilization of ΔΨ_m_, leading to cell death was demonstrated by Zorov et al. (2000). This was followed by studies by Aon et al. (2003) who provided strong evidence for the involvement of IMAC as a mediator of RIRR in metabolic oscillations. Pharmacological studies confirmed that IMAC facilitates superoxide release (Takahashi and Asada, 1983; Paky et al., 1993), providing the mechanism by which this anion channel contributes to RIRR. TSPO antagonists such as 4′-chlorodiazepam and PK11195 inhibit anion transport by IMAC, consistent with a strong modulatory role of TSPO on this ROS-sensitive channel (Beavis, 1989; Kinnally et al., 1993). In response to stress, IMAC activation occurs first, ultimately followed by mPTP activation at higher stress levels (Aon et al., 2007; Motloch et al., 2015). Collectively, these are the key elements in the series of events leading up to ROS-induced cell death. Although brief perturbations in ΔΨ_m_ may not influence cell survival to a large extent, prolonged periods of ΔΨ_m_ instability are known to mediate mitochondrial dysfunction and cell death (Marchetti et al., 1996; Zamzami et al., 2005).

The relevance of metabolic oscillations to electrophysiological behavior was examined using photo-induced oxidation of cardiomyocytes. These seminal studies demonstrated that cyclical oscillations of the action potentials (AP) were generated in phase with ΔΨ_m_ oscillations. AP recovery was found to depend upon ΔΨ_m_ recovery, and this suggested a profound mitochondrial control of myocyte excitability, at least *in vitro* (Aon et al., 2003). More recently, we and others (Zhou et al., 2014; Alleman et al., 2016) examined the relationship between ΔΨ_m_ stability and arrhythmogenesis in response to oxidative stress. AP oscillations were generated by “out-of-phase” oscillations of sarcolemmal K_ATP_ channels during RIRR (Aon et al., 2003). Furthermore, the opening of sarcolemmal K_ATP_ channels may give rise to the phenomenon of “metabolic sink”, whereby conduction wavefronts are hindered when they encounter heterogeneous current sinks in the tissue. These current sinks are formed in regions having high open probability of sarcolemmal K_ATP_ channels (Akar et al., 2005; Akar and O’Rourke, 2011; Zhou et al., 2014). The testing of the anti-arrhythmic effects of K_ATP_ channel inhibition using glibenclamide resulted in conflicting results, including reports of adverse effects (del Valle et al., 2001), whilst the pro-arrhythmic effects of channel activation have been demonstrated by multiple groups (Fedorov et al., 2011; Xie et al., 2015). In our studies sarcolemmal K_ATP_ channel inhibition using glibenclamide did not prevent the initiation of reperfusion arrhythmias in the *ex vivo* perfused guinea pig heart (Akar et al., 2005). This highlighted the necessity for a better understanding of the upstream elements such as TSPO which could potentially modulate the deleterious opening of sarcolemmal K_ATP_ channels during RIRR.

Consistent with cellular studies of RIRR, we demonstrated that exposure of intact hearts to high doses of exogenous pro-oxidants such as H_2_O_2_ provoked two distinct ROS peaks. While the initial low amplitude peak coincided with the exogenous stressor, the second (large amplitude) peak (which we termed P2) occurred following not during the exogenous stress, consistent with a RIRR response (Biary et al., 2011). Functionally, hearts that exhibited P2 were prone to ventricular fibrillation, whereas those that did not were relatively more protected (Biary et al., 2011). In a subsequent study, we investigated the relationship between the stability of the mitochondrial membrane in response to oxidative stress and the pro-arrhythmic potential of guinea pig hearts (Xie et al., 2014). Specifically, we modulated the threshold and rate of decline of the mitochondrial membrane potential in response to exogenous pro-oxidant challenge using a variety of agents that affected the activity of key mitochondrial ion channels. Once again, hearts that exhibited rapid ΔΨ_m_ decline were associated with low thresholds for sustained arrhythmias (Xie et al., 2014). More recently, Alleman et al. (2016) elegantly demonstrated that the stabilization of the mitochondrial membrane potential may underpin exercise-mediated protection against reperfusion arrhythmias.

In light of studies showing that TSPO blockade was highly effective in abolishing ΔΨ_m_ instability, we and others examined the impact of TSPO inhibition on arrhythmia propensity. Indeed, TSPO inhibition protected against ischemia-induced AP duration (APD) shortening and inexcitability (Akar et al., 2005). In contrast, IMAC activation using the TSPO agonist FGIN-1-27 enhanced APD shortening and promoted conduction failure under ischemic conditions (Akar et al., 2005). In these hearts, high-resolution optical AP mapping revealed areas of conduction block, which gave rise to sustained re-entrant arrhythmias upon reperfusion. In contrast, TSPO inhibition protected against ischemia-induced conduction block and reperfusion-related arrhythmias. Highlighting the role of TSPO as a chief mediator of post-ischemic arrhythmias, Brown and colleagues observed similar anti-arrhythmic effects of TSPO blockade in a rabbit model of ischemia-reperfusion injury, which were not apparent in those hearts treated with the mPTP blocker, CsA (O’Rourke, 2000; Aon et al., 2003; Brown et al., 2008). In addition to pharmacological inhibition of TSPO, cardiac-specific knockdown of this gene also proved to be protective against reperfusion arrhythmias in spontaneously hypertensive rats (Ilkan et al., 2018). Ongoing studies will help determine if this novel cardiotropic TSPO gene silencing approach may have a role in combatting oxidative stress-related arrhythmias in the heart.

## Tspo in Diabetic Pathophysiology

The use of TSPO ligands in a variety of experimental settings has led to their translation to clinical trials for treatment of neurological and psychiatric diseases (Rupprecht et al., 2010). The utility of these ligands in metabolic diseases, however, has been the subject of very few investigations. A notable exception is an elegant study by Gut et al. (2013) in which treatment of zebrafish larvae with 4′-chlorodiazepam or PK11195 caused a marked decrease in systemic glucose levels, suggesting a potential role for treatment of diabetic complications. The compounds were also found to be activators of a fasting-like energy state, protecting obese mice from the undesirable effects of metabolic dysregulation (Gut et al., 2013; Gut, 2015). Other groups studied the effects of pharmacological manipulation of TSPO on adipocyte functions. Since adipose tissue is a vital integrator of glucose homeostasis, it plays a major role in the pathophysiology of metabolic diseases including diabetes (Rosen and Spiegelman, 2006). The Papadopoulos laboratory postulated that TSPO in adipose tissues could serve as a pharmacological target in the treatment of type-2 diabetes mellitus (Li and Papadopoulos, 2015). To that end, they demonstrated the efficacy of two separate TSPO ligands in improving glucose uptake and adipogenesis through TSPO activation (Li and Papadopoulos, 2015). These authors argued that the anti-diabetic effects of these ligands are mediated via modulation of mitochondrial function, and in particular, cholesterol transport thereby improving biogenesis of the lipid bilayer (Li and Papadopoulos, 2015). Of note, TSPO expression is reduced in adipocytes from obese and diabetic mice and humans compared to those from their healthy non-diabetic counterparts (Arner et al., 2010; Thompson et al., 2013; Li and Papadopoulos, 2015). The significance of these observations was underscored by genetic knockdown studies. In particular, TSPO depletion in adipocytes led to impaired glucose uptake and adipogenesis. These findings are consistent with the notion that TSPO plays a critical role in the maintenance of normal adipocyte homeostasis (Li and Papadopoulos, 2015).

Recent evidence also indicates that mitochondrial cholesterol buildup may be a key step in disease progression (Paradis et al., 2013; Musman et al., 2017). Paradis et al. (2013) reported that reperfusion of ischemic rat myocardium is linked with an accumulation of mitochondrial cholesterol, which in turn, causes the generation of oxysterols via oxidation of cholesterol by ROS. Interestingly, 4′-chlorodiazepam inhibited cholesterol accumulation and mitochondrial injury through oxysterol formation (Paradis et al., 2013). These findings revealed a novel mechanism of TSPO-related mitochondrial dysfunction that is distinct from RIRR. This alternative mechanism has been hypothesized to be of particular relevance to hypercholesterolemia, a hallmark of type-2 diabetes mellitus (Musman et al., 2017). Indeed, elevated cholesterol levels are well-known risk factors for various cardiovascular diseases including thrombosis and cardiac ischemia-reperfusion injury. Moreover, there is substantial evidence for exacerbation of cardiac injury (Hoshida et al., 1996; Scalia et al., 2001; Osipov et al., 2009), and defective cardioprotective pathways in hypercholesterolemic and diabetic conditions (Bouhidel et al., 2008; Peart and Headrick, 2009; Ferdinandy et al., 2014; Wu et al., 2014). In a follow-up study, Musman et al. (2017) demonstrated enhanced oxysterol formation in a standard rat model of type-2 diabetes mellitus. Remarkably, 4′-chlorodiazepam inhibited cholesterol transfer into mitochondria and reduced oxysterol buildup, reinstating oxidative phosphorylation and preventing mPTP opening (Musman et al., 2017). Therefore, the inhibition of cholesterol uptake by 4′-chlorodiazepam may represent a potential therapeutic strategy against ischemia-reperfusion injury in diabetes mellitus and other metabolic diseases. Preliminary work by our lab examined the role of TSPO ligands in post-ischemic arrhythmogenesis of the diabetic heart (Hu et al., 2016). In a rat model of obesity and type-2 diabetes mellitus, in which we and others found that classically cardioprotective pathways targeting mitochondria are generally impaired, we verified the effectiveness of TSPO inhibition by 4′-chlorodiazepam in protection against these arrhythmias. Future studies employing genetic knockdown and over-expression strategies are needed to better understand the role of TSPO in the electrophysiology of the diabetic heart both at baseline and in response to oxidative stress.

While the focus of this article is on the role of myocyte TSPO expression in arrhythmogenesis through the regenerative process of RIRR, mechanisms by which TSPO can contribute to sudden death is likely to be multi-factorial and not merely restricted to this phenomenon. Indeed, TSPO is expressed in numerous cell types and not just myocytes. In fact there is substantial evidence of robust TSPO expression in the endothelium, vascular smooth muscle cells, adipose tissue, platelets, and macrophages (Veenman and Gavish, 2006; Bird et al., 2010; Li and Papadopoulos, 2015; Hellberg et al., 2018). Of note, because TSPO expression in non-myocyte populations (namely macrophages) increases markedly during inflammation, TSPO serves as powerful biomarker in diabetes mellitus, atherosclerosis and other inflammatory diseases in an ever-growing number of PET studies (Pugliese et al., 2010; Hellberg et al., 2018; Ran et al., 2018). In addition to serving as a biomarker of inflammatory disease, TSPO actively participates in the regulation of non-myocyte cellular functions that likely influence arrhythmia vulnerability. For example, in macrophages, genetic and pharmacologic TSPO inhibition reduces cellular lipid content and prevents foam cell formation during atherogenesis (Taylor et al., 2014). Use of specific ligands also suggested an interesting role for TSPO in mediating white and brown adipose tissue homeostasis, pointing to its potential as a therapeutic target in the metabolic syndrome (Thompson et al., 2013). Interestingly, both epicardial adipose tissue and macrophages secrete inflammatory adipokines and cytokines which can induce structural and electrical remodeling of the myocardium (Mazurek et al., 2003; Melo et al., 2004; Iacobellis et al., 2005; Abed et al., 2013; Francis Stuart et al., 2016; Samanta et al., 2016). This provides a plausible link between non-myocyte TSPO activity and arrhythmogenesis, especially in the setting of diabetes mellitus.

## Conclusion and Future Directions

A growing body of evidence highlights the role of oxidative stress as a major mediator of arrhythmias in the setting of metabolic diseases such as diabetes (Anderson et al., 2009; Duicu et al., 2016; Peiro et al., 2016; Zhang et al., 2017). Glucose fluctuations in diabetic patients promote excessive production of ROS (Saito et al., 2014; Wu et al., 2016), which can supersede the protective antioxidant defense systems that normally operate in healthy myocardium. This leads to oxidative stress and mitochondrial dysfunction, and is often regarded as a hallmark feature of the diabetic heart. Mitochondrial dysfunction gives rise to and exacerbates numerous cardiovascular complications including endothelial dysfunction (Higashi et al., 2009), atherosclerosis (Harrison et al., 2003), myocardial infarction (Misra et al., 2009), and diabetic cardiomyopathy (Jia et al., 2018), all of which can lead to sudden cardiac death (Zipes and Wellens, 1998). In addition, the pathological phenomenon of RIRR is a major mediator of oxidative stress-driven cellular electrical dysfunction and death (Zorov et al., 2000; Aon et al., 2003, 2006, 2007). This process generates ROS endogenously as a response to elevated ROS levels. Given its well-characterized links to ROS-releasing mitochondrial channels, TSPO has emerged as a key hub in the regulation of mitochondrial function and the cardiac response to oxidative stress. Our understanding of the role of TSPO in diabetes will expand by combining insights gained from pharmacological and genetic studies targeting this critical outer mitochondrial membrane protein in the diabetic heart.

## Author Contributions

Both authors have contributed to the drafting, writing, and final editing of this article.

## Conflict of Interest Statement

The authors declare that the research was conducted in the absence of any commercial or financial relationships that could be construed as a potential conflict of interest.
